# Heterologous Expression of *SvMBD5* from *Salix viminalis* L. Promotes Flowering in *Arabidopsis thaliana* L.

**DOI:** 10.3390/genes11030285

**Published:** 2020-03-07

**Authors:** Yunhe Cheng, Lili Cheng, Qingchang Cao, Junzhu Zou, Xia Li, Xiaodong Ma, Jingjing Zhou, Feifei Zhai, Zhenyuan Sun, Yanping Lan, Lei Han

**Affiliations:** 1 Research Institute of Forestry, Chinese Academy of Forestry, Beijing 100193, China; heyuncheng666@163.com (Y.C.); junzhuzou010@163.com (J.Z.); lixia2530@163.com (X.L.); civsmg@163.com (X.M.); jingjingzhou0929@163.com (J.Z.); sunzy@caf.ac.cn (Z.S.); 2 Beijing Academy of Forestry and Pomology Sciences, Beijing 100093, China; lily980101@126.com (L.C.); bj51503317@163.com (Q.C.); 3 Key Laboratory of Tree Breeding and Cultivation, State Forestry Administration, Beijing 100193, China; 4 College of Agriculture and Bioengineering, Heze University, Heze 274000, China; 5 School of Architectural and Artistic Design, Henan Polytechnic University, Jiaozuo 454000, China; lkyzff@163.com

**Keywords:** *Salix viminalis*, SvMBD5, transgenic, flowering, demethylation

## Abstract

Methyl-CpG-binding domain (MBD) proteins have diverse molecular and biological functions in plants. Most studies of MBD proteins in plants have focused on the model plant *Arabidopsis thaliana* L. Here we cloned *SvMBD5* from the willow *Salix viminalis* L. by reverse transcription-polymerase chain reaction (RT-PCR) and analyzed the structure of SvMBD5 and its evolutionary relationships with proteins in other species. The coding sequence of *SvMBD5* is 645 bp long, encoding a 214 amino acid protein with a methyl-CpG-binding domain. SvMBD5 belongs to the same subfamily as AtMBD5 and AtMBD6 from Arabidopsis. Subcellular localization analysis showed that *SvMBD5* is only expressed in the nucleus. We transformed Arabidopsis plants with a *35S::SvMBD5* expression construct to examine SvMBD5 function. The Arabidopsis *SvMBD5*-expressing line flowered earlier than the wild type. In the transgenic plants, the expression of *FLOWERING LOCUS T* and *CONSTANS* significantly increased, while the expression of *FLOWERING LOCUS C* greatly decreased. In addition, heterologously expressing *SvMBD5* in Arabidopsis significantly inhibited the establishment and maintenance of methylation of *CHROMOMETHYLASE 3* and *METHYLTRANSFERASE 1*, as well as their expression, and significantly increased the expression of the demethylation-related genes *REPRESSOR OF SILENCING1* and *DEMETER-LIKE PROTEIN3*. Our findings suggest that SvMBD5 participates in the flowering process by regulating the methylation levels of flowering genes, laying the foundation for further studying the role of SvMBD5 in regulating DNA demethylation.

## 1. Introduction

Methylation of cytosine (5mC) is a conserved epigenetic modification in plants and vertebrates [1,2,3]. DNA methylation plays important roles in development and stress responses by regulating gene expression, transposon silencing, and X chromosome inactivation [1,4,5,6,7]. In mammals, 5mC is mainly present in CG dinucleotides, whereas in plants 5mC occurs in CG, CHG, and CHH (where H is A, C, or T) contexts [3,8,9]. The establishment, maintenance, and removal of methyl groups in these three sequence contexts are implemented via different pathways [2]. In plants, RNA-directed DNA methylation (RdDM) is the main pathway of DNA methylation establishment [2,10,11]. The cytosine-DNA-methyltransferases METHYLTRANSFERASE 1 (MET1) and CHROMOMETHYLASE 3 (CMT3) are key proteins for maintenance of CG and CHG methylation, respectively [1,2,12,13]. The methyltransferases DOMAINS REARRANGED METHYLTRANSFERASE 2 (DRM2), CHROMOMETHYLASE 2 (CMT2) and DECREASE IN DNA METHYLATION 1 (DDM1) are responsible for CHH methylation through the RdDM pathway [14,15,16]. REPRESSOR OF SILENCING 1 (ROS1), TRANSCRIPTIONAL ACTIVATOR DEMETER (DME), DEMETER-LIKE PROTEIN 2 (DML2), and DML3 excise 5mC from all cytosine sequence contexts [17,18,19,20,21,22].

In the classic epigenetic model, DNA methylation of promoters is responsible for transcriptional silencing [23,24]. Methyl-CpG-binding domain (MBD) proteins recognize DNA methylation and play important roles in mediating the effects of DNA methylation [25,26]. In *Arabidopsis thaliana* L., there are 13 *MBD* genes [27], which can be divided into eight subclasses [28]. Bioinformatics analysis showed that MBD5, MBD6 and MBD7 are unique to dicots [22]. Arabidopsis MBD proteins have different DNA binding abilities [28]. Only AtMBD5, AtMBD6, and AtMBD7 specifically bind to methylated CG cytosine sites [27,29]. AtMBD5 also binds to methylated CHH cytosine sites in a non-specific manner, yet it cannot bind to methylated CHG cytosine sites [25]. AtMBD6 can bind non-specifically to CHH and CHG cytosine sites [25], whereas AtMBD7 cannot bind to methylated CHH or CHG cytosine sites [25]. Other MBD proteins in Arabidopsis bind to DNA in the presence of methylated cytosines, whereas some cannot bind to DNA [25]. 

The molecular function of a protein usually depends on its structure. AtMBD7 contains three methyl-CpG-binding domains, whereas AtMBD5 and AtMBD6 each contain only one such domain [25,26,30], indicating that AtMBD5 and AtMBD6 share a closer relationship and similar functions compared to AtMBD7. AtMBD7 binds to regions of dense methylation, interacts with ROS5/IDM2 (INCREASED DNA METHYLATION 2), recruits ROS1, and ultimately participates in DNA demethylation [31]. MBD6 not only participates in RNA-mediated gene silencing in combination with 40S ribosomal protein AtRPS2C, AtAGO4, and nuclear transport factor AtNTF2, but it also binds to the histone deacetylase AtHDA6 in the RdDM pathway [32]. Studies in tomato (*Solanum lycopersicum* L.) have shown that MBD5 interacts with the CUL4–DDB1–DET1 complex, affecting its assembly on methylated DNA and thereby impairing the transcriptional activation of downstream genes [33].

The different molecular functions of MBD proteins indicate that they play different roles in plant growth and development. In Arabidopsis, *MBD* genes are expressed in a tissue-specific manner, except for *AtMBD11*, which is highly expressed in all tissues [30]. AtMBD8 and AtMBD9 participate in the regulation of flowering. *FLOWERING LOCUS T* (*FT*) and *SUPPRESSOR OF OVEREXPRESSION OF CO1* (*SOC1*) are downregulated in Arabidopsis C24 ecotype MBD8 mutant plants, which show a late-flowering phenotype [34]. However, the flowering time of mutant plants in the Col background is not delayed [34]. MBD9 regulates the expression of *FLOWERING LOCUS C* (*FLC*) by binding to various regions in this gene and binding to histone H4 [35,36]. In addition, MBD9 regulates branching, which is independent of the FLC pathway [36]. Arabidopsis MBD7-1 mutants have low *SUCROSE-PROTON SYMPORTER2* (*SUC2*) expression and develop long roots when cultivated on a sucrose-containing medium [31]. Overexpressing *SlMBD5* in tomato led to dark fruit color and dwarf plants [33]. 

To date, few studies have focused on MBD proteins in plants, especially woody plants. In the current study, we cloned the coding region of the *MBD* gene *SvMBD5* from the woody plant *Salix viminalis* L. and analyzed its expression pattern and biological function. The results provide an excellent reference for studying the functions of MBD proteins in woody plants.

## 2. Materials and Methods

### 2.1. Plant Materials

*S. viminalis* clones were provided by the willow-planting resources of the Tree Physiology Group of the Chinese Academy of Forestry. Young leaf tissue (0.1 g) was frozen in liquid nitrogen and stored at −80 °C for the cloning of *SvMBD5*. 

### 2.2. Methods

#### 2.2.1. Molecular Cloning of *SvMBD5* by Reverse Transcription-Polymerase Chain Reaction (RT-PCR)

Total RNA of *Salix viminalis* leaf tissue was isolated using an EASYspin Plus Plant RNA Kit (Aidlab, Beijing, China). A PrimeScript 1st strand cDNA synthesis kit (Takara, Japan) was used for the synthesis of first-strand cDNA from the RNA. The cDNA was amplified using specific primers (Forward: 5’-ATGTCATTGTCAGCAACTCC; Reverse: 5’-TCAACGCTTTCTGTTACCAT) designed based on the *SvMBD5* sequence obtained from transcriptome data for *Salix viminalis* L.

#### 2.2.2. Conserved Motif and Phylogenetic Analyses of SvMBD5 

MEME and Pfam programs were used for the motif analysis of SvMBD5 [37]. A phylogenetic tree was constructed based on protein sequence alignment of MBD5 proteins from *S. viminalis*, *Populus trichocarpa* L., *S. lycopersicum*, *Paeonia suffruticosa* L., *Vitis vinifera* L., *Gossypium arboretum* L., *Morus notabilis* L., *Triticum aestivum* L., *Helianthus annuus* L., and *Arabidopsis thaliana* L. with the PHYML program [38]. Phylogenetic trees of the MBD protein sequences were constructed using MEGA6 [38].

#### 2.2.3. Expression of *SvMBD5* at Different Developmental Stages

The leaves and shoot apical meristems of *S. viminalis* at the vegetative development stage (S1, May 25, 2017), floral initiation stage (S2, June 16, 2017), and floral organ development stage (S3, August 1, 2017) were sampled to detect *SvMBD5* expression by reverse-transcription quantitative polymerase chain reaction (qRT-PCR).

#### 2.2.4. Subcellular Localization of SvMBD5

**Figure 1:**
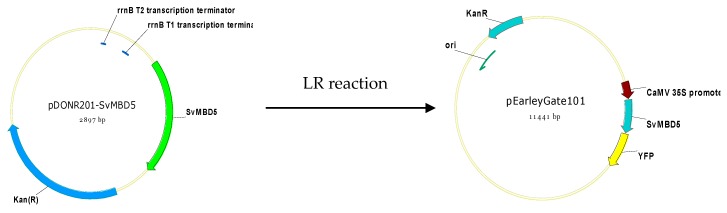
Construction of the SvMBD5-YFP expression vector.

The coding sequence (CDS) of *SvMBD5* without the terminator codon was cloned into the pEarleyGate101 vector fused with *YFP* (yellow fluorescent protein) using Gateway Technology (Life Technologies, Carlsbad, CA, USA) (Figure 1). The Gateway primers for the subcellular localization of SvMBD5 were as follows: Forward: 5’-GGGGACAACTTTGTACAAAAAAGTTGGAATGTCATTGTCAGCAACTCC and Reverse: 3’-GGCGGCCGCACAACTTTGTACAAGAAAGTTGGGTAACGCTTTCTGTTACCAT. The constructs were transiently expressed in Arabidopsis protoplasts as described by Miao et al. [39]. YFP fluorescence was imaged under a confocal laser-scanning microscope (Leica TCS SPII, Leica Microsystems, Wetzlar, Germany).

#### 2.2.5. Generation of SvMBD5-Expressing Arabidopsis Lines

**Figure 2:**
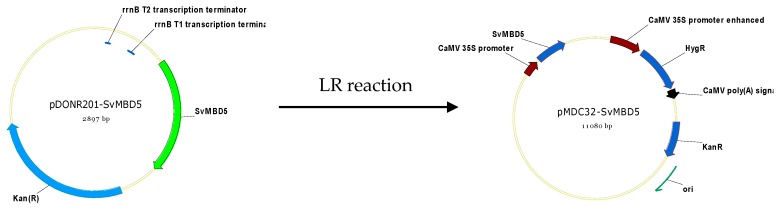
Construction of the *SvMBD5* expression vector.

The CDS of *SvMBD5* was cloned into plant expression vector pMDC32 using Gateway Technology (Figure 2). The Gateway primers for the subcellular localization of SvMBD5 were as follows: Forward: 5’-GGGGACAACTTTGTACAAAAAAGTTGGAATGTCATTGTCAGCAACTCC and Reverse: 5’-GGCGGCCGCACAACTTTGTACAAGAAAGTTGGGTATCAACGCTTTCTGTTACCAT. Arabidopsis plants (ecotype Col-0) were transformed with this construct by the floral dip method [40]. Seeds were sowed and plants were cultivated according to Wang et al. [41].

#### 2.2.6. Transgenic Plant Detection and Phenotype Analysis

Genomic DNA was isolated from Arabidopsis leaves using a MiniBEST Plant Genomic DNA Extraction Kit (TaKaRa, Japan). Transgenic plants were identified by polymerase chain reaction (PCR) analysis using the primers used for cloning. Three independent lines per construct were used for phenotypic analysis. The flowering time of *A. thaliana* was measured based on the number of rosette leaves as described by Song et al. [42]. Student’s *t*-test was used for the analysis of significant differences relative to control wild-type plants (ecotype Col-0).

#### 2.2.7. Semiquantitative RT-PCR and Quantitative RT-PCR

Total RNA was isolated from the samples using an EASYspin Plus Plant RNA Kit (Aidlab, China). First-strand cDNA was synthesized as described above (2.2.1). The expression of *SvMBD5* in Arabidopsis was detected by semiquantitative RT-PCR, and *ACTIN8* expression was used as the internal control. The semiquantitative RT-PCR primers were listed in Table 1.

SYBR Premix Ex Taq II Kit (TaKaRa, China) was used for reverse-transcription quantitative PCR (qRT-PCR) on a Light Cycler 480 system (Roche Applied Science, Penzberg, Germany) [41]. The qRT-PCR primers (Table 1) for *SvMBD5* were designed using Primer3 (http://primer3.ut.ee/). *SvACT* (*SapurV1A.0018s0700*) was used as the reference gene. The qRT-PCR primer sequences (Table 1) of flowering genes (*CO*, *FT*, *FLC*) and DNA methylation genes (*DME*, *DML2*, *DML3*, *DRM2*, *MET1*, *RDM1*, *ROS1*) in *Arabidopsis thaliana* were obtained from the qPrimerDB database (https://biodb.swu.edu.cn/qprimerdb).

## 3. Results

### 3.1. Identification and Sequence Characteristics of SvMBD5

**Figure 3:**
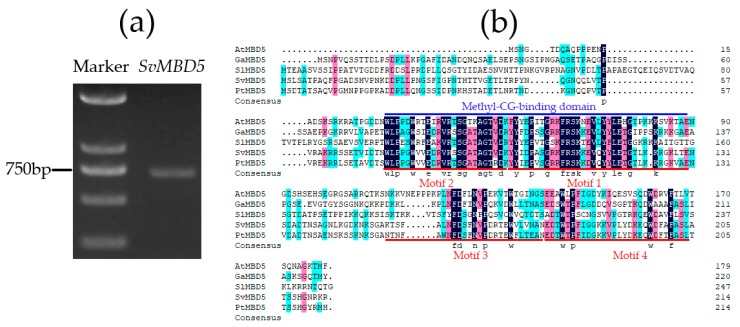
Agarose gel electrophoresis of the polymerase chain reaction product, and amino acid sequences of MBD5 proteins. (**a**) The amplification product of *SvMBD5*. (**b**) Amino acid sequence alignment of SvMBD5, GaMBD5 (*Gossypium arboretum* L.), SlMBD5 (*Solanum lycopersicum* L.), PtMBD5 (*Populus trichocarpa* L.), and AtMBD5 (*Arabidopsis thaliana* L.). The amino acid sequence of SvMBD5 was predicted according to the sequenced results of the amplification product.

We cloned and identified full-length sequences of the coding regions of putative *MBD5* homologs from *S. viminalis* (Figure 3a). The CDS of *SvMBD5* was 645 bp long (Supplementary Materials S1), encoding a 214 amino acid protein (Figure 3b). The molecular weight and theoretical pI of *SvMBD5* was 23,627.20 Da and 9.32, respectively.

**Figure 4:**
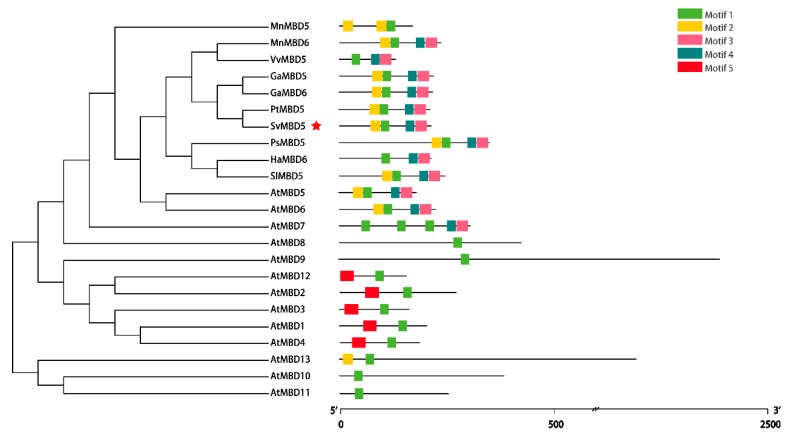
Phylogenetic relationships and motifs of MBD5 proteins in different plants.

A phylogenetic tree was constructed based on the sequences of 24 MBD proteins from nine plant species for analysis of the phylogenetic relationships among MBD homologs (Figure 4). The result showed high levels of conservation between MBD5 and MBD6 in different species. SvMBD5 belongs to the same subfamily as AtMB5 and AtMBD6 and shares the closest evolutionary relationship with PtMBD5. Sequence comparisons with other MBD homologs were performed to validate the identification of SvMBD5 (Figure 3b and Figure 4). There is one conserved methyl-CpG-binding domain in SvMBD5. We detected five motifs among all MBD proteins (Figure 4 and Table 2). Most MBD5 proteins from the nine species contained highly conserved motifs (motif 1, motif 2, motif 3, and motif 4) (Figure 4). Motif 1 and motif 2, which were found in the methyl-CpG-binding domain, are located at the N-termini of MBD proteins. Motif 1, which was the core area of the conserved MBD domain, was discovered in all MBD proteins (Figure 4). Motif 3 and motif 4 were located in the C termini of various MBD proteins (Figure 3b), specifically all MBD5 and MBD6 subfamily members. Motif 5 was only present in AtMBD1, AtMBD2, AtMBD3, AtMBD4, and AtMBD12.

### 3.2. Expression Analysis of SvMBD5

**Figure 5:**
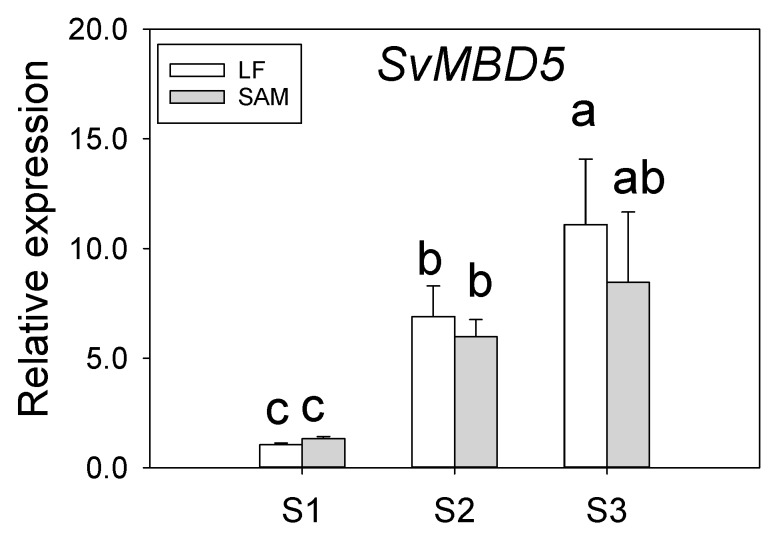
The expression of *SvMBD5* in leaves (LF) and shoot apical meristems (SAMs) in *S. viminalis* L. at different developmental stages. Stage I (S1) is the vegetative development stage. Stage II (S2) is the floral initiation stage. Stage III (S3) is the floral organ development stage. Least significant difference (LSD) tests were used to determine significant differences between samples (mean ± standard error; *n* = 3). Different lowercase letters in each column indicate a significant (*p* < 0.05) difference between samples.

We investigated the expression of *SvMBD5* in the leaves and shoot apical meristems (SAMs) of plants at different developmental stages (Figure 5). The expression of *SvMBD5* in leaves and SAMs exhibited similar dynamic changes during development. *SvMBD5* was expressed at relatively low levels during vegetative development, and its expression increased significantly at the floral initiation stage and floral organ development stage. These results suggest that SvMBD5 might play important roles in floral initiation and development.

### 3.3. Subcellular Localization of SvMBD5 Protein

**Figure 6:**
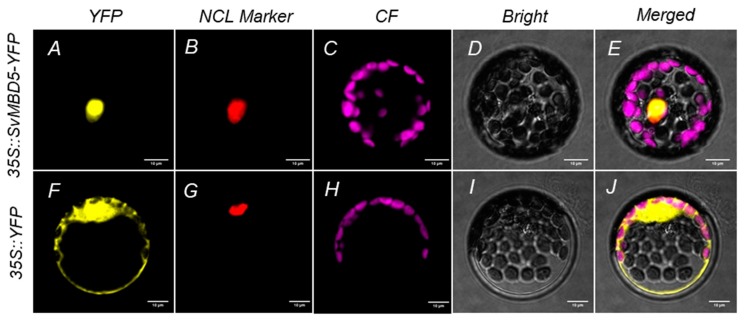
Subcellular localization of YFP-SvMBD5 in *Arabidopsis thaliana* L. protoplasts. (**A**–**E**) and (**F**–**J**) show the subcellular localization of SvMBD5 and 35S::YFP in Arabidopsis protoplasts. RedDot1 was used as the nucleus (NCL) marker. CF represents chloroplast autofluorescence.

To determine the subcellular localization of SvMBD5, we constructed a YFP-SvMBD5 fusion expression vector, transiently transformed it into Arabidopsis protoplasts, and observed the expression of YFP. Yellow fluorescence from the empty 35S::YFP vector was distributed in the nucleus and cell membrane (Figure 6F,J), while yellow fluorescence from the 35S::SvMBD5-YFP recombinant expression vector was only observed in the nucleus (Figure 6A,E). These results indicate that SvMBD5 protein localizes in the nucleus.

### 3.4. Heterologous Expression of SvMBD5 Promotes Flowering in A. thaliana

**Figure 7:**
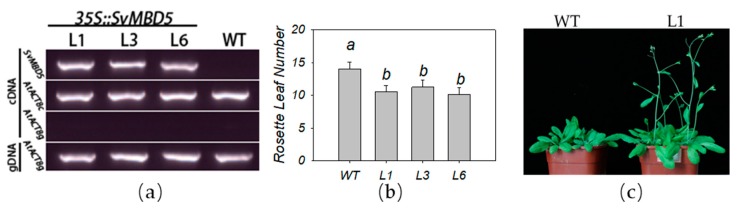
The effect of expressing *SvMBD5* on flowering time in *Arabidopsis thaliana* L. (**a**) Semiquantitative RT-PCR analysis of *SvMBD5* expression. *AtACTIN8* (*At1g49240*) was used as the internal control. Two pairs of primers (AtACT8c and AtACT8g) were used to amplify *AtACTIN8*. The amplification product of the AtACT8c primers overlaps with the first intron of *AtACT8*. The amplification product of the AtACT8c primers does not contain any introns. The two pairs of primers were used as an indicator of DNA contamination. (**b**) Analysis of flowering time in transgenic and wild-type *A. thaliana* L. (mean ± standard error; *n* = 12). Different lowercase letters in each column indicate a significant (*p* < 0.05) difference between samples analyzed by Student’s *t*-test. (**c**) The flowering phenotypes of *A. thaliana* L. plants. The transgenic plants in the photograph are from line L1.

We subjected three transgenic lines (L1, L3, and L6) to phenotypic analysis. We analyzed the expression of *SvMBD5* in the transgenic lines by semiquantitative RT-PCR using *AtACTIN8* as an internal reference gene. The expression levels of *SvMBD5* in transgenic lines L1, L3, and L6 were similar to that of *AtACTIN8* (Figure 7a). The flowering time of all three transgenic lines was significantly earlier than that of wild-type Arabidopsis (Figure 7b). These findings suggest that expression of *SvMBD5* promotes flowering in Arabidopsis (Figure 7c).

### 3.5. Expression of Key Flowering-Related Genes in SvMBD5 Transgenic Plants

**Figure 8:**
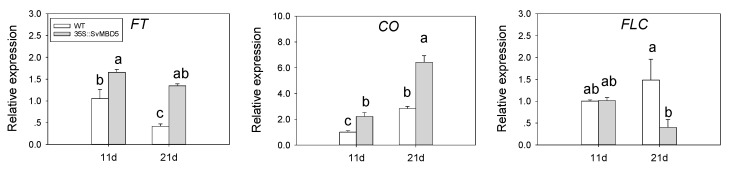
The expression patterns of key flowering genes in transgenic and wild-type Arabidopsis. Least significant difference (LSD) tests were used to determine significant differences between wild type and transgenic plants. Data are mean ± SE, *n* = 3. Different lowercase letters in each column indicate a significant (*p* < 0.05) difference between samples.

*FLC*, *CONSTANS* (*CO*), and *FT* play important roles in flowering regulation. To investigate the molecular mechanism by which *SvMBD5* promotes flowering, we measured the expression levels of key flowering genes in the leaves of Arabidopsis plants heterologously expressing *SvMBD5* (Figure 8). In the *SvMBD5* transgenic plants, the expression levels of *FT* and *CO* were higher than those of wild-type Arabidopsis at 11 and 21 days after sowing. By contrast, the expression level of the flowering inhibitor *FLC* was significantly lower in the transgenic plants than in the wild type, but only at 21 days after sowing.

### 3.6. Expression of DNA Methylation-Related Genes in SvMBD5 Transgenic Plants

**Figure 9:**
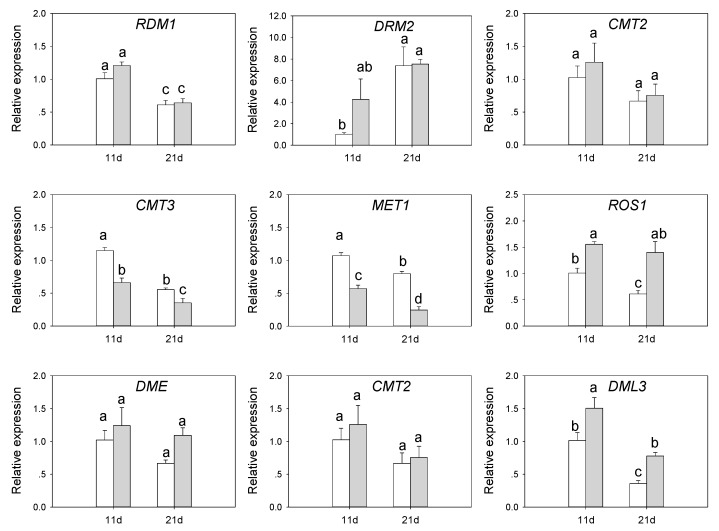
The expression of methylation-related genes in transgenic and wild-type Arabidopsis. Least significant difference (LSD) tests were used to determine significant differences between groups. Data are mean ± SE, *n* = 3. Different lowercase letters in each column indicate a significant (*p* < 0.05) difference between samples.

Plant flowering is often accompanied by large changes in DNA methylation. To investigate the relationship between *SvMBD5* and DNA methylation status, we examined the expression of key genes involved in DNA methylation establishment (*RDM1*, *DRM2*), maintenance (*CMT2*, *CMT3*, *MET1*), and removal (*DME*, *ROS1*, *DML2*, *DML3*) by quantitative RT-PCR (Figure 9). In *SvMBD5*-expressing plants, the expression levels of the DNA methylation establishment-related genes *RDM1* and *DRM2*, as well as the DNA methylation maintenance gene *CMT2*, appeared to be higher than those in wild-type Arabidopsis at 11 and 21 days after sowing, but these differences did not reach significant levels. However, the expression levels of *CMT3* and *MET1* in *SvMBD5*-expressing plants were lower than those in wild-type plants. The expression levels of *CMT3* and *MET1* in transgenic vs. wild-type Arabidopsis were significantly different (*p* < 0.05), indicating that expressing *SvMBD5* significantly inhibited the expression of *CMT3* and *MET1*. Among the DNA methylation removal-related genes, the expression levels of *DME* and *DML2* (associated with demethylation) appeared to differ slightly between transgenic and wild-type Arabidopsis, but these differences were not significant. The expression levels of *ROS1* and *DML3* in transgenic Arabidopsis were significantly (*p* < 0.05) higher than those in wild-type Arabidopsis. These results indicate that expressing *SvMBD5* significantly increases the expression of *ROS1* and *DML3*.

## 4. Discussion

### 4.1. MBD5 Might Be Involved in Demethylation

DNA methylation is one of the most important epigenetic modifications in plants. MBD proteins function as interpreters of DNA methylation. In Arabidopsis, AtMBD5, AtMBD6, and AtMBD7 share a relatively close relationship. Furthermore, MBD5 and MBD6 belong to the same subfamily, as both of these proteins contain only one methyl-CpG-binding domain. However, MBD7 contains three methyl-CpG-binding domains. The different molecular structures of these proteins might contribute to their functional differences. AtMBD7 participates in active demethylation by specifically binding to methylation-dense sites, and they cooperate with ROS5/IDM2 to recruit ROS1 [31]. MBD6 binds to the histone deacetylase AtHDA6 in the RdDM pathway [32]. In the current study, heterologously expressing *SvMBD5* significantly increased the expression of *ROS1* and *DML3* in Arabidopsis; these genes function in the active demethylation pathway. In *A. thaliana*, four bifunctional 5mC DNA glycosylases, ROS1, DME, DML2, and DML3, can excise 5mC from all cytosine sequence contexts [17,18,19,20,21,22]. Our results suggest that SvMBD5 might be involved in activating the demethylation of DNA via ROS1 and DML3.

In addition, heterologously expressing *SvMBD5* significantly reduced the expression of *CMT3* and *MET1* genes which function in the methylation maintenance pathway. *MET1* is a homolog of the DNA methyltransferase gene *DNMT1* in mammals and is responsible for maintaining the methylation of CG sites [1,43]. MET1 is also involved in the establishment of DNA methylation in the RdDM pathway in plants [14]. CMT3 can bind to H3K9me2 at CHG sites to maintain CHG methylation [12,44]. This finding suggests that SvMBD5 might also prevent the maintenance of CG and CHG methylation. ROS1 can antagonize the RdDM pathway and prevent DNA methylation [45], suggesting that DNA methylation and demethylation are synergistic processes. These findings indicate that SvMBD5 can promote DNA demethylation but inhibit DNA methylation and methylation maintenance. This finding not only highlights the antagonism of demethylation on the RdDM pathway, but also indicates that the DNA demethylation pathway inhibits the pathway for maintenance of DNA methylation. Our results provide a powerful reference for further revealing the relationship between active DNA demethylation and DNA methylation maintenance.

### 4.2. How Does the Heterologous Expression of SvMBD5 Promote Flowering?

MBD proteins have different effects on plant growth and development. In Arabidopsis, the MBD8 mutant exhibits delayed flowering and reduced *FT* and *SOC1* expression, whereas the expression of *FLC* is not significantly altered in this mutant [34]. Arabidopsis *MBD9* plants show early flowering and significantly reduced *FLC* expression. The early flowering phenotype of this mutant was eliminated by overexpressing *FLC* [35]. These findings suggest that MBD8 and MBD9 affect flowering via different pathways. However, Arabidopsis plants showed no significant phenotypic changes when the expression of *AtMBD6* and *AtMBD7* was inhibited [27]. Therefore, AtMBD5, AtMBD6, and AtMBD7 may share functional redundancy. In the current study, heterologously expressing *SvMBD5* in *A. thaliana* promoted flowering. In the transgenic plants, *FLC* was significantly downregulated compared to wild-type plants, whereas *FT* and *CO* were significantly upregulated. These results indicate that SvMBD5 promotes flowering by influencing the expression of flowering genes (*FT* and *CO*) and a flowering inhibitor gene (*FLC*).

DNA methylation levels tend to increase in plants during the transition from vegetative to floral stages [46,47,48,49]. In Arabidopsis plants heterologously expressing *SvMBD5*, demethylation genes (*ROS1*, *DML2*) were upregulated and methylation maintenance and establishment genes (*CMT3* and *MET1*, respectively) were downregulated, suggesting that SvMBD5 promotes flowering by relieving or inhibiting the methylation of flowering-related genes. However, a previous study examining whole-genome DNA methylation patterns during flower development in Arabidopsis did not detect significant changes in methylation in *FT*, *CO*, or *FLC* [47]. Moreover, Finnegan demonstrated that DNA methylation is not directly involved in regulating *FLC* expression in the vernalization pathway [50]. These studies suggest the SvMBD5 does not directly regulate the expression of *FT*, *CO*, and *FLC*. However, a recent study showed that the loss of methylation in the *CONSTANS-LIKE2D* homolog *COL2D* was associated with its higher expression levels and promoted flowering in cotton (*Gossypium hirsutum*) [51]. The different results between Arabidopsis and cotton indicate that the role of DNA methylation in flowering regulation varies among species.

## 5. Conclusions

The CDS of *SvMBD5* is 645 bp long and encodes a 214 amino-acid protein with one MBD domain. *SvMBD5* belongs to the same subfamily as *AtMBD5* and *AtMBD6* in Arabidopsis. SvMBD5 localizes in the nucleus. Heterologously expressing *SvMBD5* in Arabidopsis reduced the expression of the flowering inhibitor gene *FLC*, increased the expression of flowering genes *CO* and *FT*, and promoted flowering. *SvMBD5* increased the expression of *ROS1* and *DML3* in the demethylation pathway and inhibited the expression of *MET1* and *CMT3* in the methylation establishment and maintenance pathway. These findings suggest that MBD5 is involved in the DNA demethylation pathway.

## Figures and Tables

**Table 1:** Primers used for reverse transcription-polymerase chain reaction (RT-PCR) and reverse-transcription quantitative polymerase chain reaction (qRT-PCR).

	Gene	Left Primer (5’→3’)	Right Primer (5’→3’)
RT-PCR	*AtACT8c*	CTGGAAAGTGCTGAGGGAAG	GGACTCTGGTGATGGTGTGT
	*AtACT8g*	ACCACCAATCCAGACACTGT	TCCACATGCTATCCTCCGTC
	*SvMBD5*	TCAACGCTTTCTGTTACCAT	ATGTCATTGTCAGCAACTCC
qRT-PCR	*SvMBD5*	CTGTCCATCCACCTTCCCTT	TCCCGAGTCTGAAGGAGAGA
	*SvACT*	AGAGGACTTCAGGACAACGG	TTTCACAACCACAGCAGAGC
	*ACTIN8*	CTCCAGCGAATCCAGCCTTA	GCCGATGCTGATGACATTCA
	*CMT2*	CCGAGCATACATTGTCGAAATT	CGCTTAATTGTAATTCGCCTGA
	*CMT3*	TTCCCTCTTCCAACTTGACTAC	GATTGAGAAAGGATGAAGCGTC
	*CO*	CCATGGATGAAATGTATGCGTT	GGAGATAGAGTTGTTCCGCTTA
	*DME*	CAGCAGTTCTATCTCTCGGTAG	ACTCCTCTGAAGAATGCCTTAC
	*DML2*	AGTTCTGATTGCTACGTGAGAA	CTAGCATAAACCCTATCGACGT
	*DML3*	CTTGGGGAAGTTTACACAATCG	GTTGACACAAATGTTGGTCGTA
	*DRM2*	GATGAAACACACTTCTCCACAC	ACATCAAAGGTGTAGGAGAAGG
	*FLC*	GGAGATTTGTCCAGCAGGTGA	GCCAAGAAGACCGAACTCATG
	*FT*	TGACAATTGTAGAAAACTGCGG	CTACAACTGGAACAACCTTTGG
	*MET1*	CGACAATCATAATCCGCCAATT	CTGATGTTGAAGATCGTCCAAC
	*RDM1*	ATACATCTCTGCTCTTCTCAGC	CAATGACAATGGAACTACGACC
	*ROS1*	TAACAGGAGTTACAGGCACAAT	TGAACTTTGGAAACGACGTAAC

**Table 2:** The motifs in methyl-CpG-binding domains (MBDs).

Motif Name	Sequence	Length (aa)	Annotation
Motif 1	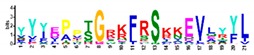	21	_
Motif 2	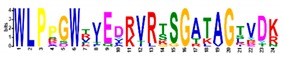	24	Methyl-CpG-binding domain (PF01429)
Motif 3	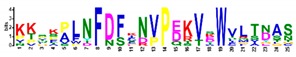	25	_
Motif 4	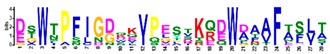	28	_
Motif 5	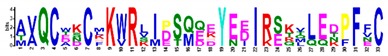	33	_

## Supplementary Materials

The following are available online at https://www.mdpi.com/2073-4425/11/3/285/s1, Supplementary Material S1: The coding sequence of *SvMBD5*.
