# Supplementary material for: Heterologous Expression of SvMBD5 from Salix viminalis L. Promotes Flowering in Arabidopsis thaliana L
Source: Genes (Basel). 2020 Mar 7;11(3):285. doi: 10.3390/genes11030285 (PMC7140845; doi:10.3390/genes11030285)
Supplement: Supplementary file 1 [file genes-11-00285-s001.pdf]

The coding sequences of *SvMBD5*:
ATGTCATTGTCAGCAACTCCGGCTCAATTTCCGGGTGCGGACTCGCATGTACCCAA
TAAAGATGATCCCCTCCTTCCAAACGGATCATTTATCGGCCCCAACACCCACACC
ACCGTCGGTGAAACCCTAACCAGACCTTACAACCAAGGCAATCAACAACTGGTG
ACGCCTGTTCGAGCGAAGAGAAGATCATCAGAAACGGTCATTGATACGAATTGGT
TGCCGCCGGGATGGGTTGTTGAAGATCGGGTTAGGAGCTCTGGTGCGACAGCTGG
CACCGTAGATAAGTATTACATTGATCCTGCCTCAGGTCGCAAGTTCAGATCCAAGA
AAGAGGTCCAGTATTATTTGGAAACTGGGACTTTAAAGAAGAGGGGGAAATTGA
CAGAGAACTCTGATGCTGACACTAATTCTGCAGGGAATTTAAAGGGCGATAAAAA
CAAGTCTGGCGCAAAAACAAGTTTTGCTTTGAACTTCGATTCTTTTAATGTTCCTG
ATAGGACCGAATGGGTTCTTGTAAATGCAAATGAAGATACGTGGACTCCCTTCATT
GGTGGTAAAAAAGTGCCTCTATACGATAAGGAACAATGGGATTTTGCTTTTGCATC
TCTGGCGACAAGCAGCCATGGTAACAGAAAGCGTTGA
